# 

**Published:** 1881-02

**Authors:** 


### INTERNATIONAL MEDICAL CONGRESS.

An international Medical Congress, with a Section for diseases of the Teeth, will be held in London, England, beginning August 2d and continuing till the 9th, inclusive, 1881.

On the general programme this constitutes Section 12.

The following list of subjects have been prepared for consideration at that meeting:

1st. Replantation and transplanting of teeth.

2d. Premature wasting of the alveoli, and its amenability to treatment.

3d. The share taken by septic agents in causing disease of the dental pulp and periosteum.

4th. Mercurial and syphilitic teeth, and the causes of irregularities of position of the teeth.

5th. New dental instruments and methods of operating.

Other topics may be introduced if time and opportunity will permit.

This will be an occasion of great interest and importance to the dental profession throughout the world. It is gratifying that in preparing and arranging for an international medical congress, dentistry has been assigned a position, and dentists invited to co-operate in the proceedings of the occasion.

A full and complete organization for this Section has been made by the profession in London, and of such a character as to insure success.

Mr. Edward Saunders, Esq., has been elected President of this Section, and Mr. C. S. Tomes, Esq., F. R. S., No. 37 Cavendish Square, W., Secretary.

The President and Secretary request all who intend to be present to notify them as early as possible, also to give any suggestions in regard to subjects for discussion.

A contribution of one guinea (or five dollars) will be required from those who are to be members, at the time of taking out their Cards of Admission. This will constitute membership, will admit to all the meetings, and will entitle each member to a copy of the Transactions of the Congress.

Such contributions may be sent to the President or Secretary. This, with notice of intention to be present, should be sent at an early day.

### ALUMNI MEETING.

The third annual meeting of the alumni of the Ohio College of Dental Surgery will be held in the lecture room of the College on Thursday, March 3d, 1881.

It is desirable that all the graduates of the institution be present so far as possible, and add to the interest of the occasion, and especially should all the members of the Association be present.

The Executive Committee have in preparation an interesting programme, the carrying out of which, all will heartily enjoy.

A. Berry,

N. S. Hoff,

F. W. Sage,

} *Ex. Committee.*

The Annual of the Ohio Dental College Association will be held in the College, on Tuesday, March 1st, at 2 o'clock, P. M. It is very desirable that all members be present.

### MARRIED.

On December 2d, 1880, Dr. L. G. Noel, D. D. S., of Nashville, Tennessee, to Miss Nannie Folwell, of Memphis, Tenn.

The happy pair will have the warm congratulations and best wishes of all their friends and acquaintances. May their happiness increase and grow brighter throughout that voyage upon which they have just entered

### CONTENTS.

### COMMUNICATIONS.

### THE DENTAL REGISTER,

A MONTHLY MAGAZINE DEVOTED TO THE INTERESTS OF

**THE DENTAL PROFESSION.**

*TERMS REDUCED TO $2.50 PER YEAR. SINGLE COPIES 25C.*

EDITED BY PROF. J. TAFT, D.D.S.

AND

*Published by SPENCER, CROCKER & CO., Ohio Dental Depot.*

The volume commences in January and closes in December.

The REGISTER being issued monthly is always fresh, therefore Indispensable to the practitioner who desires to keep posted up with the new inventions and improvements which are daily developed. Neither expense nor effort will be spared to give value and variety to its contents. Articles will be illustrated with well executed engravings whenever they will add to the interest or assist to explanation.

Its extensive circulation and frequency of issue make it one of the BEST DENTAL ADVERTISING MEDIUMS in the United States.

All subscriptions must begin with January or July.

Local or special notices, five lines or less, will be inserted for $3 each insertion; over five lines, 50 cts. per line.

All advertising bills payable in advance, or at furthest, after the issue of the first number containing the advertisement. All transient advertisements to be paid for in advance.

☞ CONTRIBUTIONS SOLICITED.

Exclusively Editorial Communications should be addressed to Editor.

The latest number of the REGISTER may be ordered with or without other goods at any time, and all letters should be addressed to

***SPENCER, CROCKER & CO., Ohio Dental Depot, Cincinnati, O.***

